# How physicians make sense of their experience of being involved in hospital users’ complaints and the associated mediation

**DOI:** 10.1186/s12913-019-3905-8

**Published:** 2019-01-28

**Authors:** Béatrice Schaad, Céline Bourquin, Francesco Panese, Friedrich Stiefel

**Affiliations:** 1 Communication office, Lausanne University Hospital, BU21/03/284/, Rue du Bugnon 21, 1001 Lausanne, Switzerland; 2 Psychiatric Liaison Service, Lausanne University Hospital, Lausanne, Switzerland; 3 Faculty of Social Sciences and Politics, Lausanne University, Lausanne, Switzerland

**Keywords:** Patient complaints, Complaints center, Physician-centered research, Lived experience, Interpretative phenomenological analysis

## Abstract

**Background:**

The growing interest in hospital users’ complaints appears to be consistent with recent changes in health care, which considers the patient’s voice a valuable information source to improve health care. Based on the assumption that the clinicians’ lived experience is an essential element of health care and to neglect it may have serious consequences, this study aimed to explore how physicians experience hospital users’ complaints and the associated mediation process.

**Methods:**

A qualitative analysis of experience narrative interviews. Fourteen physicians concerned by complaints which resulted in a mediation provided a comprehensive narrative of their experience with the complaints center. Data were analyzed with Interpretative Phenomenological Analysis (IPA). Interviews were analyzed inductively and iteratively to explore how physicians make sense of their experience.

**Results:**

The analysis of the physicians’ narratives revealed that being the object of a complaint and to enter a process of mediation is a specific experience of which some physicians benefited and others felt psychologically weakened. The causes of the complaints were at times considered by physicians to be related to medical malpractice, but more often to communicational and relational difficulties, unrealistic expectations of patients, physicians’ attitudes, or the lack of a coherent care plan. The analysis of their narratives revealed that physicians showed a need for reconsidering and elaborating on the reason(s) leading to the complaint, and on the expectations patients/relatives may have had towards medicine and health care professionals. This may be interpreted as an attempt to assign their meaning, such meaning having the potential to ease the distress associated with the experience of complaints.

**Conclusion:**

Most physicians appeared more aware of the communicational and relational aspects of care after experiencing a complaint situation; however, prior to the complaint, physicians seem to have underestimate these issues, and when they acknowledge that the complaint originated in psychological aspects of care, they still consider it not relevant, since not related to clinical decision-making and management. Mediation as providing the opportunity to restore the clinical relationship should be encouraged at an institutional level as well as support of health care professionals by means of individual or group supervision.

## Background

The potential of complaints to improve health care has long been underestimated, be it because of the tendency to conceive complaints as failures and therefore conceal or ignore them [1, 2], or to attribute complaints to an individual – a patient or staff member – instead of considering them symptoms of systemic malfunctions [3, 4]. The growing interest in hospital user complaints appears to be consistent with recent changes in health care, which increasingly acknowledges the importance of patient satisfaction, and considers the patient’s voice a valuable information source to improve health care [5–15]. Hospitals have developed several tools to measure patient satisfaction over recent decades: satisfaction survey results are for instance commonly viewed as valuable data, even though their sensitivity tends to be overestimated [4]. This lack of sensitivity calls for a monitoring system of patients’ complaints, which provide information on problems not captured by surveys: “patients and families, collectively, observe a huge amount of data points within healthcare settings; they have privileged access to information on continuity of care, communication failures, dignity issues […]; they are outside the organization, thus providing an independent assessment that reflects the norms and expectations of society” [15]. Greater consideration of complaints also results from the judicialization of medicine, namely the increasing tide of medical-malpractice litigation, and its consequences for litigants (patients and health care professionals), such as psychological suffering or defensive medicine [16–18]. This is reflected in recent attempts to seek alternative dispute resolutions (e.g., mediation) [4, 6, 8, 19] or new ways of gathering and handling complaints, for instance by means of complaints center for hospital users [4, 19–23].

In a previous study, we examined the content of complaints collected in a complaints center [24] and showed that the main reason for patients, their relatives, and friends to visit the center was related to the interpersonal relationship with health care professionals; this type of complaints was significantly more frequent than those concerning technical aspects of care. The present study focused on another person involved in the complaint. It aimed to explore how *physicians* experience hospital users’ complaints and the associated mediation process. This “physician-centered” study was based on the assumption that the clinicians’ lived experience is an essential element of health care and to neglect it may have serious consequences, such as psychological suffering, dissatisfaction and effects on the way medicine is practiced [25, 26].

While literature on complaints filed with a hospital complaints center hosting professional mediators is rather sparse [27, 28], physicians’ attitudes towards legal complaints have been investigated [29]. This study was to our knowledge a first exploration of the physicians’ lived experiences of a complaint handled in the hospital.

## Methods

The research design was a qualitative approach of experience narrative interviews with physicians using Interpretative Phenomenological Analysis (IPA) [30].

### Setting

In April 2012, Lausanne University Hospital, Switzerland (a 1471-bed inpatient facility with about 47,000 yearly admissions) opened a complaints center for patients, their relatives, and friends [24]. In the *Espace Patients & Proches* (EPP), three professionals trained in mediation (i) provide space for voicing concerns and dissatisfaction; (ii) document and examine the health care process underlying complaints; and (iii) help to improve the quality of care and services. Complainants can request mediation with the concerned health care professional(s), who can consent or not to participate (the vast majority of them consent). At the time of the study (2015), among a total of 467 complaints 5% lead to a subsequent mediation process.

From its opening to September 2018, The EPP has dealt with 3000 complaint situations.

### Participants

The mediators were asked to identify all complaints concerning physicians that resulted in a mediation process since 2012; 25 situations met this criterion. Since complaints are handled confidentially, a stepwise protocol was developed to contact physicians: the mediators contacted physicians, informed them of the study purpose, and obtained their agreement to be contacted by an investigator not employed by the EPP (CB). Of the 25 identified physicians, 20 agreed to be approached by CB, 3 refused to participate owing to lack of time, and 2 could not be reached, since they did not respond to phone or email. Of the 20 remaining physicians, 14 took part in the study; the others did not follow-up the interview request or were no longer available owing to overwork. The final study sample, then, pragmatically included physicians who participated in an interview (*N =* 14).

The professional background of the 5 female physicians and 9 male physicians participating in the study includes notably gynecology, psychiatry, geriatrics, surgery, and adult internal medicine; 6 were chief residents, 4 senior staff physicians, 3 residents, and one was head of service. It was for all physicians the first time to face a complaint involving a third party.

### Accessing the experience and attitudes of physicians

To draw as close as possible to the meaning physicians attribute to their experience of patient complaints, in-depth interviews were conducted. The format could be termed “experience narrative interview”. Physicians were encouraged to recount their experience, i.e., the situation leading to the complaint and how they reacted and felt about it. If they did not spontaneously address the following topics, the investigator introduced them (interview schedule): individual and professional/clinical impact of the complaint, ways of dealing with the complaint process, and perceptions of the EPP. The interviews lasted 18–52 min and were audiotaped and transcribed verbatim.

### Data analysis

Data analysis was conducted inductively and iteratively using IPA [30]. IPA is a qualitative research approach developed by Smith et al. [30], which aims (i) to try to understand and describe study participants’ world, and (ii) to provide an interpretation analysis of the “personal sense-making activities” of the participants [31]. Based on the transcripts, two authors, BS and CB, developed third-person narrative descriptions, which had to closely reflect and cohere with physicians’ views and attempted to highlight the elements structuring their thoughts and experiences. Next, a comprehensive data exploration, through the interview topics, was conducted to obtain an understanding of the physicians’ world (provisional meanings). The last analytical steps were more interpretative (e.g., “what it means” for the physicians to have expressed these perceptions, views, feelings) and focused on physicians’ meaning and sense-making [31]. Emergent themes were developed and searched for patterns and structure, and a “heuristic narrative” was produced to provide an overview of the analysis. Finally, interview excerpts were selected to illustrate the themes and give voice to the participants.

BS and CB conducted a parallel analysis of the third-person narrative descriptions, discussed the analysis in depth, and reached agreement on interpretation. A dialogue was fostered with the other authors (FS [a psychiatrist] and FP [a sociologist]) to develop and enhance the coherence and plausibility of the interpretation.

## Results

Results of the analysis of the 14 interviews are first reported here by means of the “heuristic narrative” to show how themes – i.e., what matters for physicians when they make sense of their experience of patients/relatives’ complaints – tend to be connected for the participants as a whole. This heuristic narrative is an interpretative synthesis of the analytic work. A detailed description of the emerging themes is then provided, with excerpts from the interviews.

### Physicians’ sense-making of their experience: An overview narrative

Physicians showed a need for reconsidering and elaborating on the reason(s) leading to the complaint, and on the expectations patients/relatives may have had towards medicine and health care professionals [theme: *What happened?: attributed causes*]. This may be interpreted as an attempt to assign their meaning, and even “truth”, to what they had to face; such meaning having the potential to ease the distress associated with the experience of a complaint. Their decision to disclose or not to disclose this experience to their colleagues and/or superiors reveals the importance attributed to being subjected to a complaint and to its clinical relevance [theme: *To share or not to share*]. In this respect, the fact that communication-related complaints seemed to be considered problems unrelated to clinical decision-making and management is especially interesting [theme: *To change or not to change*].

Secondly described is the complaint experience with regard to the relationship physicians usually have with their patients, resulting in a seeming definition of what they perceive to be a good relationship [theme: *What is a good patient relationship*]*.* Their perception, in turn, influenced if the complaint was considered as comprehensible or not and the associated feelings (e.g., relief about the mediation when the relationship was viewed as tense or sadness when the therapeutic alliance was considered as having been strong) [theme: *How it affected me*]. When complaining patients/relatives were experienced as unpredictable, the meaning physicians were able to derive from the experience was hampered; the complaint was related to a specific type of person rather than a situation [theme: *The complainant*]. Finally, the mediators were difficult to apprehend by the physicians because of a lack of knowledge of their role (particularly their neutral stance) and of the aim of mediation [theme: *The mediation*].

Physicians contextualized their experience by referring to the role and missions of the EPP, and to the practice of medicine nowadays. With respect to the EPP, physicians considered its role to achieve an overview on the patient’s trajectory*,* to allow patients/relatives to express themselves, to reduce tensions outside the clinical setting, to provide responses patients did not receive from health care professionals, and to serve as a supportive third party which – particularly in difficult situations – assisted them in a non-judgmental way: *“I told myself, well there is an arbitrator who will tell us how to communicate”* ^*(3 = number of the interview)*^. Some physicians also felt that the EPP contributes to the improvement of the quality of medical services by reporting on the managed cases: *“It is important that information from the EPP is fed back to the medical services, otherwise one has no opportunity to improve, to reflect.”* ^*(14)*^. Others, however, considered that the EPP took over the health professionals’ duty to manage problematic situations (particularly owing to lack of time or objectivity), possibly judged the physicians’ behaviors*: “A little bit like pointing the finger or something like that?”* ^*(6)*^, or wasted the physician’s time: *“Because it comes to discussing and not to punishment* [of the guilty ones, whoever they are]*”* ^*(11)*^*,* generating more work for health care professionals and even encouraging patients against whom health care professionals already have to defend themselves*: “We have to defend ourselves against people who are never satisfied, because they pay too much insurance and have the impression they also have more rights and so on”* ^*(11)*^*.* Finally, the EPP was sometimes considered as “promoting a culture of complaints” and tarnishing the hospital’s image: *“When one goes to a very good restaurant, if there is a vomitorium just next door, […] I think it gives a bad impression.”* ^*(11)*^

The broader context of medical practice was also addressed, such as medicine being omnipotent: *“Medicine wants to solve everything. With this type of medicine, error is not possible*” ^*(7)*^, or the power of litigating patients who take their frustration out on physicians: *“The philosophy now in our hospital is essentially that every time a patient behaves in an unbearable manner, one writes a letter saying that we are sorry that everything didn’t turn out as well as we would have liked to, instead of saying ‘Listen Mister, you behaved in an absolutely inappropriate manner […] so you can stick your letter you know where’*” ^*(11)*^*.* Similarly, some physicians felt that the hospital sacrificed physicians in fear that patients may turn to the media, and that university hospitals are “*trash cans*” ^(11)^ obliged to treat all patients, including those with a reputation of being difficult and litigious.

### Emergent patterns of meaning

#### What happened?: Attributed causes

Physicians considered that most complaints originated from communicational or relational difficulties ^(1, 2, 3, 4, 5, 6, 7, 8, 10, 12, 13)^, such as questions that remained unanswered ^(3, 5, 13)^, a lack of mutual understanding ^(3,6)^, or an unbalanced relationship ^(2)^. But they also conveyed that complaints issued from malpractices and neglect ^(1, 7, 8, 9, 10, 12, 13, 14)^, such as a missed diagnosis ^(10)^, the non-respect of medical confidentiality ^(7)^, an unsuccessful operation ^(1)^, the lack of a care plan or of advance directives^(6)^. Physicians also considered that the complaint was related to characteristics of themselves, like being incompetent ^(3)^ or lacking pro-activity ^(6)^*: “What was hardest to endure* [for the patient] *was me”* ^(12)^.

They furthermore explained the patient’s/relatives’ decision to turn to the EPP as a consequence of their tendency to perceive physicians as seemingly inaccessible to hear a complaint; as if they were on a pedestal ^(4, 14)^. The involvement of the EPP was also interpreted as a mean of patients to have an effect (e.g., to destabilize physicians by taking them out of the clinical setting ^(2)^, to fight against fragmentation of care by bringing together all health care professionals involved in their care ^(7)^), and as an expression of a deception about medicine. Complaints were thus considered to emerge from situations in which medicine was unable to solve the problem of the complainant ^(1, 3, 6, 9, 13, 14)^. Here, physicians particularly referred to complaints related to non-acceptance of a chronic disease ^(14)^, the rapid evolution of a life-threatening illness ^(1)^, the progression of a problem (e.g., senile dementia) considered as reversible by relatives – *“The patient had to be helped to eat […] she [his wife] came and said: ‘Listen, my husband is getting worse, there is a problem, as physicians you must solve this problem, I have to get my husband back, as before’”* ^*(3)*^ – or the impression that the medical staff had not done everything possible ^(6)^, and that a medical intervention (e.g., in vitro fertilization) had failed ^(13)^.

#### To share or not to share

Physicians informed their hierarchy or colleagues about the complaint ^(2, 3, 4, 6, 8, 9, 10, 12, 13, 14)^, either because they wanted to convey their perception of the situation ^(2)^ or because the complaint was considered as being “just” related to a “communication problem”^(4)^ and thus – unlike an error in clinical judgment – of little importance. Other physicians decided to not inform their hierarchy for the very same reason: because they attributed the complaint to communication issues and thus without further consequence ^(4, 12)^. These attitudes may indicate that physicians conceived communication problems and associated complaints as minor issues without a clinical impact.

#### To change or not to change

The estimated impact of the complaint process on practice seems to validate the last interpretation; indeed, physicians felt that its main effect was on communication ^(2, 3, 4, 6, 8, 10, 12)^: for example, increased repetition of information ^(6, 8)^, verifying that information is understood ^(6, 8)^, or acknowledging that a lack of communication is bad communication ^(3)^. Physicians also reported changes in how they started to explore patients/relatives experience of the situation, and to listen carefully, particularly if they were passive, anxious, or suffering: *“Maybe I should have listened more to the fact she was really anxious about her [medical] history”* ^*(10)*^.

#### What is a good patient relationship

Physicians made sense of the complaint experience in light of what they defined as a good relationship and good communication with patients/relatives, such as referring patients when there were communication problems ^(1, 5, 11, 13)^, or addressing the issues of death and the limits of medicine ^(3)^. This can be interpreted as an attempt to demonstrate and defend their way of encountering patients/relatives and practicing medicine. They emphasized for instance the importance of being transparent by explaining treatment protocols and their benefits ^(3, 5, 6, 7, 8)^, by initiating immediate discussions with the family when a complication or *“a silly thing”* ^*(5)*^ occurs, and by *“taking responsibility for all the shit”* ^(11)^ and fixing resulting problems*.* Physicians also stressed the importance of responding to patient/relative complaints, for example, by not avoiding the situation, accepting mediation, and dealing with threats from patients who wanted to write to the hospital management ^(1, 5, 11, 13)^, and they mentioned their willingness to encounter the patient as a person: *“I always try to ask myself… what is the person like?”* ^*(3)*^*.* Finally, physicians endorsed the need for reflexivity ^(3, 4, 11)^, particularly by constantly questioning oneself and by learning to accept criticism: *“A resident or a chief resident who can’t bear to be criticized misses his vocation”* ^(11)^. Good medical care was also evoked, characterized by the ability to recognize, as a physician, the limits of medicine, which may involve ceasing rather than increasing investigations or providing detailed information to prevent unrealistic expectations ^(3, 14)^.

#### How it affected me

The emotional experience associated with the complaint process was influenced by how physicians perceived themselves as “carers” and how they conceived their relationship with the complainant(s).

Physicians expressed *sadness* ^(1, 2, 4, 10, 12, 13)^: *“[…] I was also depressed because they did not recognize all I have done for them”* ^*(13)*^ or *surprise* ^(2, 3, 4, 6, 9, 10, 12, 13, 14)^ that patients had contacted the EPP or that a stranger (the mediator) had informed them of the problem: *“To me, it was like out of the blue, to hear this, not from my usual staff”* ^*(3)*^*.* They were also surprised that patients/relatives had turned their tragedy into a complaint ^(9)^, or considered the provided care as a bad experience ^(12)^. Some reported a certain *satisfaction*^(1, 2,)^ that they gained through this experience a capacity to understand that hospital users undergo subjective experiences, that their reproach is legitimate, and that they benefited from the procedure initiated by the EPP. *Relief* ^(2, 4, 7, 8, 10, 11, 12, 13)^ was experienced due to the ending of the relationship with the complainant or to the involvement of the EPP as a neutral third party. *Fear* and *apprehension* ^(2, 4, 6, 7, 9, 13)^ were felt regarding conflict evolution: *“It begins like this but I don’t know how far it could go”* ^(2)^, having made a mistake, or the perception of a threatening attitude of the complainants.

Feelings of *discomfort* ^(2, 3, 4, 9, 12, 14)^ were mostly associated with a tendency to take the complainant’s criticism personally or to blur the professional and personal spheres: *“As physicians, as caregivers, it is extremely difficult to retain a distinction between one’s caregiver position and one’s own self”* ^*(3)*^. *Self-criticism* ^(2, 3, 8, 12, 13)^ was caused by doubts about one’s capacities: *“Did I make mistakes with everybody or only with her?”* ^*(2)*^ Physicians also related feelings of “*being a punching ball*” ^(2)^, feelings that they had to shoulder all the blame, and that they lack support from mediators and the hospital: *“Mediators do not have to support physicians even if physicians would sometimes like to be supported* ^*(4)*^*.”*

#### The complainant

The sense conveyed here is that the complaint was related to the profile of the patients/relatives rather than the clinical situation, which implies that physicians were unable to act on the situation that led to the complaint. Complainants were thus characterized in terms of their attitude ^(1, 2, 3, 4, 6, 7, 8, 9, 11, 12, 13, 14)^, their way of being in the world ^(2, 3, 4, 7, 8, 11, 14)^, their emotional state ^(1, 3, 4, 7, 8, 9, 12, 13, 14)^, and their individual characteristics ^(1, 2, 3, 4, 7, 8, 9, 11, 12, 13, 14)^, including their origins, rather than in relation to what they reported of having experienced. They were described as anti-physician: *“The son in particular felt an animosity towards the medical community ‘Anyhow, you protect each other’”* ^*(4*)^, malicious: *“When she saw I was affected and crying, it was as if it provided her with a kind of satisfaction”* ^*(12)*^, judgmental ^(3)^, and unable to accept apologies and listen to explanations ^(12,14)^. Regarding their way of being in the world (i.e., their relationships with themselves, others, and the world), complainants were viewed as difficult to please ^(3, 7, 14)^, self-centered ^(14)^ or persecuted and persecuting ^(7)^, and their emotional states was described as *“vengeful”*^*(9)*^*, “desperate”*^*(13)*^*, “depressed”* ^*(13)*^*, or “in need of reassurance”* ^*(8)*^*.* Finally, physicians described complainants by means of personality traits linked to origins (African ^(12)^, not French-speaking ^(13)^), cultural and socio-demographic background (Moroccan’s emotionalism ^(13)^, advanced age ^(13)^, modest income ^(13)^), psychic fragility (paranoid, psychotic, drug-dependent) or biography (history of abuse) ^(3, 4, 13)^.

#### The mediation

Physicians did not have a shared perception of the mediators and their role. Some physicians evoked their neutrality and acknowledged that mediators who are not involved in the situation can be more objective ^(1, 3, 4, 10, 12, 13)^, gain perspective ^(1, 3, 6, 10)^, and “smooth things”: *“It was a good thing to have a mediator who was framing the discussion […] There was such an emotion, such a revolt, such a rage, we needed somebody”* ^*(1)*^. For others, neutrality was interpreted as passivity: *“He did say he would do something for us, if we wanted, but I got the impression it would be only a little bit [he was just like] an observer”* ^*(3)*^. While some physicians felt that the mediators supported them and served health care professionals (e.g., because they marshaled complaints) ^(1, 3, 4, 6, 7, 9, 10, 12, 13, 14)^, others considered mediators to be the complainants’ defenders^(4)^ or to lack lucidity regarding the complainant’s personality or the subject of the complaint ^(12)^.

Mediation as such was considered as allowing the recognition of the experience of the complainants ^(1)^, the improvement of the relationship with them, and mutual concession, or as generating frustration: *“Difficult patients stay difficult”* ^*(11)*^.

## Discussion

The complaint and the associated mediation process were positively perceived when considered supporting patients/relatives *and* physicians, because of the opportunity provided to all to express themselves, to be recognized, and to re-establish a relationship [32, 33]. The causes of the complaints were at times considered to be related to medical malpractice, but more often to communicational and relational difficulties, unrealistic expectations of patients, physicians’ attitudes, or the lack of a coherent treatment plan. Consequently, patients/relatives motivations to complain were not perceived as searching for justice and compensation, but as attempts to provide a testimony of their experience, to affect the concerned physician, or to influence the way medical care is provided.

The complaint situation impacted on physicians’ emotions and thoughts, and lead to changes in behavior and practice. The emotional experience ranged from satisfaction and relief to feelings, such as guilt, surprise, frustration, sadness, irritation and fear, often linked to the perception of not having been recognized by patients/relatives for one’s efforts. This last impression, to feel not recognized, resonates with the motivation of patients to file a complaint [34]: the two parties thus shared a desire to be recognized, which is the source of intense emotions by all concerned persons. Some physicians appear to be capable to benefit from the experience, which increased their willingness to reflect on the situation, whereas others reacted with self-accusations or, on the contrary, a defensive stance. Contrasting attitudes were reported when it comes to sharing or withholding that one has been the object of the complaint: both attitudes were guided by the impression that communicational difficulties with patients lack relevance, since they were not considered as having a negative impact on clinical judgment. Furthermore, communication difficulties were not perceived as part of clinical care; this is erroneous since studies show that: “[…] it is not the quality of the medical care, the quality of the chart documentation, and negligent treatment per se that leads to litigation, but, rather, ineffective communication with patients” [35].

As a consequence of the complaint and mediation experience, physicians reported that they have modified the way they encounter patients/relatives, indicating an increased sensitivity towards communicational and relational aspects of care. However, prior to the complaint, physicians seem to have underestimate these issues, and when they acknowledge that the complaint originated in psychological aspects of care, they still consider it not relevant, since not related to clinical decision-making and management.

A limitation of this study may be the relatively modest sample size, but IPA aims at examining a phenomenon in depth, and not at generating a generalizable theory.

While most physicians acknowledge the positive role a hospital complaints center can play, they also express ambivalence, and report collateral negative effects they are subjected to. By providing the opportunity to restore the relationship between health care professionals and patients/relatives, mediation can be a constructive alternative to the litigation process, and should be further researched.

Considering that communication and interpersonal relationship were often viewed as causing the complaints, support for health care professionals by means of individual or group supervision, or similar approaches should be encouraged at an institutional level. Such an offer may prevent conflicts and stimulate reflection on situations which provoke suffering and negative feelings.

## Conclusions

Compared with the resulting defensive medicine after legal complaints, the impact of a hospital complaint seems to be less dramatic. For some physicians, however, the complaint experience may also have negative consequences, and support should be considered. While there might be patients/relatives who have difficulties to accept the limits of medicine, and who takes out their frustration on the physician, physicians also have to accept that what counts is often not facts, but their subjective interpretation. In some of the physicians’ narratives, patients/relatives appeared in an unflattering light, and their reference to psychopathology or sociodemographic attributes might indicate a desire to “fight back”, with some physicians considering themselves as victims of an unfriendly environment. The institution was here viewed as not supportive enough towards those who try to give their best. The hospital appears thus not only a place of nurture and care, but also – be it for hospital users or health professionals – a place of suffering where the patient and the clinician sometimes feel not recognized as a person. We have to care for the patient, but we also have to hear the voices of the physicians, as illustrated by this study, and to respond to their needs.

## Abbreviations

EPP: Espace patients&Proches
IPA: Interpretative Phenomenological analysis
